# Testing a systematically braided alcohol reduction and HIV status neutral intervention among people receiving STI care in Malawi: study protocol for a pilot hybrid type 1 effectiveness-implementation randomized controlled trial

**DOI:** 10.3389/fpubh.2025.1572288

**Published:** 2025-04-30

**Authors:** Kathryn E. Lancaster, Agatha K. Bula, Mitch M. Matoga, Mina C. Hosseinipour, Irving F. Hoffman, Jaslyn A. Grullon, Eric Umar, Jimmy Msolola, Jessica F. Magidson, Jessica L. Bonumwezi, Judith A. Hahn, Angela M. Parcesepe

**Affiliations:** ^1^Department of Implementation Science, Wake Forest University School of Medicine, Winston-Salem, NC, United States; ^2^UNC Project, Malawi, Tidziwe Centre, Lilongwe, Malawi; ^3^University of North Carolina Chapel UNC Institute for Global Health and Infectious Diseases, Chapel Hill, NC, United States; ^4^Kamuzu University of Health Sciences, Blantyre, Malawi; ^5^Center for Substance Use, Addiction & Health Research (CESAR), University of Maryland, College Park, MD, United States; ^6^Department of Psychology, Loyola University Maryland, Baltimore, MD, United States; ^7^Department of Medicine and Department of Epidemiology and Biostatistics, University of California, San Francisco, San Francisco, CA, United States; ^8^Department of Maternal and Child Health, Gillings School of Global Public Health, University of North Carolina at Chapel Hill, Chapel Hill, NC, United States

**Keywords:** evidence based intervention, alcohol reduction, HIV status neutral, sexually transmitted infection, preexposure prophylaxis, antiretroviral therapy

## Abstract

**Background:**

Heavy alcohol use is common in Malawi among people receiving sexually transmitted infections (STI) care and is a critical barrier to the success of HIV prevention and treatment efforts.

**Methods:**

This protocol presents a pilot hybrid type 1 effectiveness-implementation trial evaluating the short-term effectiveness and implementation of a scalable evidence-based intervention (EBI) to reduce alcohol use and provide HIV prevention and treatment counseling for people with heavy drinking receiving STI care in Malawi. We developed a 3-session intervention, Treat4All, that uses motivational interviewing, problem-solving skills, psychoeducation, alcohol refusal, HIV prevention and treatment skills building, and goal setting to reduce alcohol and facilitate engagement in HIV prevention and treatment. We have also integrated HIV prevention content to focus on persistent PrEP use and HIV treatment adherence to improve antiretroviral therapy (ART) adherence and viral suppression. We will conduct a two-arm pilot randomized controlled trial (RCT) in an STI care setting in urban Malawi to compare the preliminary effectiveness and implementation of Treat4All to usual care for decreasing the proportion of heavy drinking days, corroborated with phosphatidylethanol, an alcohol biomarker, and improving HIV outcomes (viral suppression among PWH; PrEP use among those at risk). We will randomly assign 160 people receiving STI care in Lilongwe who report heavy drinking (*n* = 80 people with HIV; PWH; *n* = 80 people at high risk of HIV acquisition) to Treat4All or usual care.

**Discussion:**

Our study will produce a systematically braided, scalable HIV status-neutral EBI for alcohol reduction and optimization of HIV prevention and treatment behaviors to evaluate in a larger effectiveness-implementation trial. Our study will directly expand alcohol reduction and HIV status-neutral programs for alcohol-impacted populations throughout sub-Saharan Africa and other regions where alcohol contributes to the ongoing HIV epidemic.

**Clinical trial registration:**

ClinicalTrials.gov, NCT06668363.

## Highlights

- This study advances alcohol reduction and HIV status-neutral interventions for both PWH and those at risk for HIV.
- It targets an underserved population—individuals with heavy alcohol use in STI care—broadening the scope of HIV prevention efforts.
- Incorporating alcohol biomarkers (phosphatidylethanol, PEth) offers an objective measure to enhance accuracy in evaluating intervention efficacy.
- The research sets the stage for future hybrid effectiveness-implementation trials focused on cost-effectiveness and long-term outcomes.
- It provides critical insights for scaling and systematically braiding alcohol reduction and HIV prevention programs across sub-Saharan Africa and beyond.

## 1 Introduction

Forty years into the HIV epidemic, people living in sub-Saharan Africa (SSA), including Malawi, continue to experience a disproportionate burden of HIV infection. In Malawi, HIV prevalence among adults aged 15–49 is 6.7%, with new infections ongoing (10,000 persons/year; HIV incidence rate of 0.98/1,000 adults), and nearly 30% of people with HIV (PWH) remaining virally unsuppressed (1). Non-HIV sexually transmitted infections (STIs) are common among PWH and those at high risk of HIV and are a marker of condomless sex, multiple partners, high-risk sexual networks, increased vulnerability to HIV, undiagnosed and unsuppressed HIV, and increased viral shedding (among PWH) (2–6). In our prior work, HIV prevalence among people receiving STI care in Malawi was 22% (7). Among people with STI/HIV co-infection in the largest STI clinic in the capital of Malawi, 72% were either newly diagnosed with HIV or previously diagnosed and not on antiretroviral therapy (ART) (8). People with an STI are a priority target population for interventions to improve HIV prevention and treatment. Given this context, people with an STI represent a key population for interventions that improve HIV prevention and treatment.

Heavy alcohol use significantly contributes to STI risk and undermines HIV prevention and treatment efforts (9–11). Nearly 40% of adults in Malawi drink alcohol, with approximately half of those (47%) reporting hazardous drinking, defined as an Alcohol Use Disorders Identification Test (AUDIT) score of ≥3 for women and ≥4 for men (10, 12, 13). Between 40–50% of PWH globally have a history of heavy or hazardous alcohol use (14). Among PWH, hazardous alcohol use is associated with poor adherence to ART, resulting in poor HIV viral suppression, which may lead to opportunistic infection and death (15). Heavy alcohol use has been consistently associated with adverse HIV treatment outcomes, including delayed ART initiation, suboptimal ART adherence, and virologic failure (16–19). Among people at high risk for HIV, heavy drinking has been associated with reduced HIV testing, repeat STIs, and condomless sex, compromising the success of HIV prevention strategies (20–26). Heavy drinking may also be associated with suboptimal outcomes throughout the pre-exposure prophylaxis (PrEP) care continuum, including low PrEP awareness and interest and suboptimal adherence to PrEP (27, 28). These disruptions across the HIV care and prevention continuum pose a significant barrier to achieving the UNAIDS 95–95-95 targets, which rely on effective diagnosis, sustained treatment, and viral suppression (29). Effectively addressing heavy alcohol use among people with STIs has the potential to improve HIV prevention and treatment outcomes and reduce HIV acquisition and transmission. HIV status-neutral interventions that target alcohol reduction and HIV prevention and treatment among people with STIs may ultimately reduce HIV incidence by simultaneously increasing viral suppression among PWH and PrEP use among people at risk for HIV.

Despite the prevalence of heavy alcohol use and its detrimental impact on HIV outcomes, evidence-based interventions (EBIs) for alcohol reduction remain scarce across SSA, especially in HIV and sexual health care settings (30, 31). In 2020, only 30% of surveyed HIV clinics in SSA, and just 19% in Southern Africa, reported providing any alcohol screening or treatment. Integrating EBIs to reduce alcohol use into sexual health care settings could reach a large, high-risk population, improving both HIV prevention and treatment outcomes (12, 31–34). Malawi’s national commitment to scaling up alcohol use treatment and the recent implementation of PrEP services in 2021 make it a prime location for this work.

Project TrEAT, a Trial for Early Alcohol Treatment, is a brief (2-session), highly effective, scalable, evidence-based intervention (EBI) for alcohol use reduction (35–40). TrEAT has been successfully tested with diverse populations of PWH and has led to significant improvements in the percentage of days abstinent from alcohol and viral suppression (35, 38, 39). TrEAT has not been adapted for the shifting HIV care environment to include ART and PrEP use content for both PWH and people at high risk. To address this gap, we have developed a new, systematically braided intervention called Treat4All. This three-session intervention integrates HIV status-neutral counseling with TrEAT, focusing on problem-solving, skills-building for alcohol refusal and HIV prevention and treatment use, and goal-setting (41). Delivered through the use of motivational interviewing approaches, Treat4All aims to reduce heavy drinking and improve HIV outcomes, specifically viral suppression for PWH and PrEP use for those at high risk of HIV.

The objectives are to evaluate short-term effectiveness and implementation of Treat4All for reducing heavy drinking and optimizing HIV outcomes, specifically viral suppression among PWH and PrEP use among those at high risk at 6 months post-enrollment. Additionally, we will explore enablers and barriers of the Treat4All intervention response with responders and non-responders.

## 2 Methods

### 2.1 Trial design

We are conducting a pilot hybrid type 1 effectiveness-implementation randomized controlled trial in one STI care setting in Lilongwe to compare the efficacy of Treat4All to usual care for decreasing the proportion of heavy drinking days and optimizing HIV outcomes (viral suppression among PWH; PrEP use among those at risk). We will examine the proportion of heavy drinking days, corroborated by a random subsample using phosphatidylethanol (PEth), and successful engagement in HIV prevention and treatment at 6 months. Using mixed-methods data collection, we will also assess the acceptability, feasibility, and fidelity of the Treat4All.

### 2.2 Participants and study procedures

Our primary objective is to evaluate the short-term effectiveness and implementation of Treat4All for decreasing heavy drinking and optimizing HIV outcomes (viral suppression among PWH and PrEP use among those at a high risk of HIV). We will randomly assign 160 people receiving STI care at the Bwaila STI clinic in Lilongwe, Malawi, who reported heavy drinking (*n* = 80 PWH; *n* = 80 people at high risk of HIV acquisition), to Treat4All or usual care.

Guided by the situated Information, Motivation, and Behavior (sIMB) model, we will conduct semi-structured interviews with responders and non-responders of Treat4All 6 months post-enrollment (*n* = 30). Responders will include those who have decreased the proportion of heavy drinking days or have achieved HIV outcomes. Non-responders will be those who did not.

### 2.3 Eligibility criteria

In coordination with the STI Clinical Director, we will host clinic meetings to introduce the project and study plans to clinical patients. Patients receiving STI care will be provided with information about the project by clinic staff, and those interested will be screened by clinic staff for eligibility.

Patients receive routine opt-out HIV testing as part of their usual care (UC). To be eligible, patients must: (1) be aged 18 or older, (2) be receiving care at the Bwaila STI clinic, (3) report recent heavy drinking (≥4 drinks a day in the past 30 days for men and ≥3 drinks a day in the past 30 days for women), (4) be willing to receive an HIV test at study enrollment, (5) report not taking ART or having a recent history of unsuppressed viral load (>1 viral load test with >40 copies of HIV-1 RNA per milliliter in the past 2 years) [for PWH] *or* not currently taking a PrEP prescription (self-reported taking <1 pill per week) [for those at risk of HIV], (6) plan on residing in Lilongwe for the next 6 months, (7) not participating in other HIV or alcohol programs, (8) ability and willingness of participant to provide informed consent, and (9) willingness to provide contact/locator information to be contacted for follow-up study activities.

Patients will be ineligible if they are: (1) unable to participate in study activities due to psychological disturbance, cognitive impairment, or threatening behavior, (2) unwilling to provide locator information, (3) pregnant or breastfeeding (given that a greater understanding of alcohol use during pregnancy would be warranted but limited within the planned sample) (42, 43), (4) unwilling to receive an HIV test, (5) at risk of experiencing alcohol-related withdrawal symptoms (measured using an adapted Clinical Institute Withdrawal Assessment Alcohol Scale Revised [CIWA-AR]) (44), (6) have acute physical or mental illness, including suicidal thoughts or behaviors, (7) serious illness including tuberculosis or opportunistic infection, requiring systemic treatment and/ or hospitalization, (8) active drug dependence that in the opinion of the site investigator, would interfere with adherence to study requirements, or (9) any other condition that in opinion of the study investigator would compromise the safety of the study participant or study staff or would prevent proper conduct of the study. If they are determined to be eligible, the research staff will administer informed consent and schedule their visits. Patients will be made aware of the voluntary and confidential nature of the study. Trained research assistants will collect the required data.

### 2.4 Intervention

The intervention arm (Treat4All) will include a total of 80 participants who will be randomly allocated to Treat4All. The intervention will follow a workbook but will also be flexible to allow for discussion of the intersection of alcohol, HIV prevention and treatment, and sexual risk to emerge naturally within the context of the sessions. There will be three sessions, and the actual session content of Treat4All will be tailored to a function of an individual’s particular issues and HIV status.

Treat4All is a systematically braided intervention in which three EBIs are systematically blended based on common elements to create an integrated alcohol reduction and HIV status-neutral intervention (45). Briefly, the braiding process included five steps: (1) Initial expert consultations; (2) formative qualitative interviews (total *n* = 40; *n* = 5 STI care providers, *n* = 5 bar/bottle shop staff and owners, and *n* = 30 receiving STI care with heavy drinking, i.e., *n* = 15 with HIV and *n* = 15 at risk for HIV); (3) identification of core mechanisms; (4) initial draft of braided components; and (5) follow-up expert consultations on the initial draft. Fleming’s Project TrEAT, a Trial for Early Alcohol Treatment, was enhanced with culturally appropriate HIV status-neutral content adapted from HIV Prevention Trial Network studies 082 and 074 for HIV prevention and treatment counseling, respectively.

Guided by the situated Information, Motivation, and Behavior (sIMB) framework, which emphasizes the role of information, motivation, and behavioral skills in sustaining health behaviors, Treat4All represents a novel approach to address alcohol use and HIV in Malawi. The sIMB uses the three core determinants of initiating and sustaining behavior over time: information, motivation, and behavioral skills (41). The information determinant refers to the facts and knowledge of the behavior (41). Motivation refers to the attitudes and beliefs about the positive and negative consequences of adopting health-promoting patterns of behavior at the personal and social levels (41). Lastly, behavioral skills refer to the abilities and self-efficacy that guide the successful adoption of behavior patterns (13, 46, 47). This framework ensures that individuals have the knowledge, motivation, and skills needed to initiate and maintain health-promoting behaviors.

In addition, Treat4All leverages the AIM (Assess, Identify, Make a Plan) activity from the Life Steps behavioral activation framework, which was previously adapted for HIV prevention in HPTN 082. Completing the role of information, motivation, and behavioral skills, AIM can help empower participants to address alcohol use and HIV prevention and treatment through structured goal-setting and action planning. In this adapted AIM model, participants will be encouraged to assess their current behaviors and health status, particularly those related to alcohol use and HIV infection. They then will identify personalized goals that align, such as fewer days of drinking or initiating PrEP. Finally, they will make a plan by setting specific, achievable steps that integrate goals into daily life, supported by motivational and practical tools. This tailored approach enables participants to engage actively in their health improvement journey, reinforcing self-efficacy and supporting long-term behavioral changes in areas critical to alcohol use and HIV.

Each 30-min session, delivered over three weeks (up to 12 weeks allowed), provides personalized feedback on alcohol use, discusses its health impacts, and addresses reasons for reducing consumption. Participants will identify risky situations, learn handling strategies, and set behavioral goals. Alcohol-related content covers drinking patterns, harm, and change strategies, while the HIV component promotes ART adherence and PrEP for those at risk. This approach, using problem-solving, skills building, and goal setting, aims to reduce heavy drinking and improve HIV care engagement, fostering ART adherence, viral suppression, and PrEP uptake. The formative qualitative interviews informed the culturally tailored content, ensuring that the intervention addressed locally relevant drinking behaviors, HIV-related challenges, and effective, context-specific strategies for change. Interventionists will use a Treat4All booklet and a collaboratively developed flipchart in each session to enhance intervention fidelity, participant engagement, and comprehension. The booklet provides structured guidance to ensure consistent delivery of core content, while the flipchart serves as a visual aid, illustrating key concepts that support participant understanding. This flipchart, created with input from both US- and Malawi-based intervention teams and a graphic designer, incorporates culturally relevant and accessible visuals tailored to the goals of the intervention. For example, intervention materials include images of common Malawian drinks to illustrate the alcohol content of standard drinks. To minimize potential bias from the Chichewa translation, all intervention materials and instruments underwent translation and back-translation processes, followed by role-playing by research staff to ensure linguistic and cultural accuracy. Any discrepancies were resolved through consensus with bilingual experts to maintain consistency and avoid confounding the findings. This collaborative approach ensures that the materials are both engaging and effective in conveying essential information.

The intervention sessions will be delivered by a trained intervention counselor with a diploma in psychology and a certificate of addiction counseling in Malawi. Intervention counselors will complete a weeklong in-person training at the UNC Project in Lilongwe. Training included a comprehensive review of the interventionist training manual, booklets, and flipcharts. The interventionist training manual was developed to serve as a resource, providing an overview of the study and Treat4All intervention, educational components on alcohol-related harm and HIV prevention and treatment, the importance of ensuring patient confidentiality, skills building, and counseling techniques (e.g., motivational interviewing, problem-solving skills, and goal setting). Additionally, it includes information on self-care for interventionists to focus on reducing stress and burnout. The training also included discussions on session content flow, making any necessary revisions, and reviewing the standard operating procedures. Interventionists engaged in role-playing and empty-chair practices to enhance their session delivery skills. Role-play and empty-chair practice continued for 3 weeks, with weekly debriefs discussing successes and challenges.

All Treat4All sessions will be audiotaped, and a random subsample of 20% will be reviewed to ensure intervention fidelity to the core intervention components. A bilingual Chichewa-English speaker who has received training in both intervention manuals listens to one or two audio-recorded intervention sessions biweekly and rates the interventionist’s fidelity to the intervention. The ratings will be discussed in weekly supervision meetings.

A total of 80 participants will be randomly allocated to the UC group. UC includes sexual risk reduction counseling that integrates messaging on reducing alcohol use before sex and the recommendation to abstain from or reduce alcohol while on ART, with limited further guidance. Participants in this arm will continue to receive all STI- or HIV-related services.

All participants, regardless of the study arm, will receive counseling from a trained HIV counselor in the STI clinic as part of the standard of care. Condoms will be available in the clinic. All PWH or newly diagnosed participants will be referred to a Lighthouse Trust HIV clinic, as needed per the standard of care.

### 2.5 Effectiveness outcomes and study measures

Participants will complete enrollment and baseline data collection before being randomly allocated the assignment to one of the two study arms. Participants will receive a random assignment through the prepared envelopes to Treat4All or UC at a ratio of 1:1. Masking of participants and intervention counselors to study arm assignment in the field is not feasible because of the nature of the interventions; data collection staff, analysts, and investigators will be masked to the study assignment until analyses are complete. Participants will be followed up for 6 months (Table 1).

**Table 1:** Schedule of enrollment, interventions, and assessments.

Timepoint	Enrollment	Baseline	Allocation	Post-allocation		
	*t_0_*	*t_0_*	*t_0_*	*t_0_*	Month 3	Month 6
Enrollment						
Eligibility screen	X					
Informed consent	X					
Random group allocation			X			
Intervention						
Treat4ALL				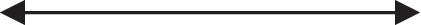		
Usual care				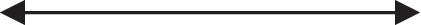		
Assessments						
Quantitative survey						
Sociodemographics and alcohol coping skills		X				
Readiness to change alcohol use Social desirability, sexual risk, HIV testing history, PrEP use history, PrEP knowledge and willingness, and ART initiation		X			X	X
HIV-related stigma, depression, health status, quality of life					X	X
Qualitative interviews (Treat4All arm only)						X
Primary outcomes						
Heavy drinking days (TLFB)		X			X	X
Engagement in HIV prevention (PrEP use) or treatment (viral suppression)		X				X
Secondary outcomes						
All participants						
PEth level^*^		X				X
STI prevalence		X				X
People with HIV only						
Viral suppression		X				X
ART adherence		X			X	X
People at risk for HIV only						
PrEP use		X				X
HIV incidence						X

^*^Dried blood spots will be collected from all participants.

The baseline questionnaire is a quantitative assessment that will be performed before arm allocation with participants by trained field staff and completed safely in a confidential place. The survey will be administered to gather information on alcohol use, including self-reported percentage of heavy drinking days measured by the timeline followback (TLFB),readiness to change alcohol use, alcohol coping skills, sociodemographics, social desirability, sexual risk, HIV testing history, PrEP use history, PrEP knowledge and willingness, ART initiation, and self-reported ART adherence. Before the start of the RCT, we will pilot the baseline survey and administration procedures with up to 40 people with similar characteristics to our target population (*n* = 20 PWH; *n* = 20 people at risk for HIV). The pilot will be assessed for survey length and checked for response distribution, item comprehension, and data-collection procedures.

Participants will also complete a series of laboratory assessments at baseline. (1) Urine samples will be collected from all participants with consent and tested for the presence of chlamydia, gonorrhea, or syphilis. (2) Blood samples will be collected from all PWH and sent to the UNC Project Malawi laboratory to assess HIV-1 RNA levels using the Abbott RealTime HIV-1. (3) Finger-stick dried blood spots (DBS) will be collected from all participants for phosphatidylethanol (PEth) testing to detect alcohol use during the 21 days before the study visit (48–50). Samples will be kept in a dry box with a humidity indicator card to monitor the moisture levels until they are brought to the laboratory. Samples will be taken to the local laboratory for storage and shipment processing. PEth is detected in whole blood by an LC–MS/MS assay using dried blood spots (United States Drug Testing Laboratories, Des Plaines, IL, USA). PEth is specific to alcohol metabolism, and slow degradation allows alcohol detection for ~21 days. PEth levels are useful for detecting changes in drinking habits within a participant over time. In 2022, the Society of PEth Research (PEth-NET) produced a 2022 Basel Consensus Document recommending a whole blood PEth cutoff concentration of 20 ng/mL as strongly suggestive of drinking more than minimal amounts (50).

All participants will be asked to complete 3- and 6-month follow-up visits. At months 3 and 6, participants will receive a quantitative follow-up survey that will gather updated information from the baseline on alcohol use, HIV testing history, health status, quality of life, social desirability, readiness to change alcohol use, social support, depression, HIV-related stigma, sexual risk behaviors, PrEP knowledge and willingness, PrEP initiation, and ART initiation and adherence. A laboratory assessment during the month 6 visit will repeat baseline collection, including urine collection for chlamydia, gonorrhea, and syphilis testing; blood sample collection from PWH for HIV-1 RNA testing; and finger-stick DBS collection for Peth testing. Additionally, all participants who tested negative for HIV at baseline will be tested for HIV following the Malawian National HIV Testing and Counseling guidelines, which indicate three serial HIV antibody rapid tests.

We will collect detailed information on alcohol use through the Alcohol Timeline Followback (TLFB) to obtain the number of heavy drinking days, percentage of days abstinent, drinks per drinking day, and measure changes in drinking over time (51). This interviewer-administered assessment reconstructs a daily behavioral calendar to prompt memory recall for alcohol consumption. TLFB has demonstrated good test–retest reliability, convergent validity, and agreement with collateral reports on daily drinking (52, 53). Specific quantities of alcohol consumed will be collected for each day for the 21 days (mirroring the consumption time frame detected by PEth) prior to the baseline, 3, and 6-month assessments. Daily alcohol consumption will be recorded as the number of standard drinks consumed. In general, and as we have previously used in Malawi, 341 mL of beer, 142 mL of wine, 34 mL of liquor/commercial spirit, a 50 mL sachet of local spirit distilled from grains, sweet potatoes, or sugar cane, a 330 mL can of fruit ale, or a 1,000 mL packet of chibuku will equal one standard drink (54–56). An alcohol biomarker will be randomly ascertained in 25% of the participants to validate self-reports at 6 months. The *proportion of heavy drinking days in month 6 will be defined as the proportion of days of heavy drinking (≥4 drinks per day for men and ≥3 drinks per day for women) in the past 21 days*. DBS samples will be collected from all participants. Based on budgetary constraints, a subsample (25%) will be randomly selected for alcohol biomarker testing, equally distributed from the Treat4All or usual care arms. This approach allows for objective measurement of alcohol use while balancing feasibility within a pilot trial context. PEth results >20 ng/mL will be defined as evidence of recent heavy drinking (50, 57, 58). Participants who self-report <50% for heavy drinking days but have PEth results >20 ng/mL will be assigned a proportion of 50% for heavy drinking days within the previous 21 days (50).

Following our theoretical premise that TrEAT will impact HIV prevention and treatment behaviors, we define our co-primary outcome of successful engagement in HIV prevention or treatment with parallel behaviors across HIV status: PrEP use for people at risk of HIV and viral suppression for PWH at 6 months. *PrEP use will be defined as a self-report of >1 pill in the past seven days a month 6. Viral suppression will be defined as an undetectable viral load (<40 copies/mL) at month 6*. Our primary outcome will not be stratified by HIV status. However, we will also assess viral suppression and PrEP use separately among PWH and those at risk for HIV, respectively, as secondary outcomes. In this way, we will assess the short-term efficacy of Treat4All among all participants (inclusive of HIV status) and separately among PWH and those at risk for HIV.

### 2.6 Retention

Based on recent HPTN clinical trials at the UNC Project-Malawi, we anticipate a 90% retention rate among participants (59). To maximize retention, the Malawi research team will apply the following procedures: (a) computerized follow-up program to track scheduled and missed study visits; (b) updating baseline locator information at a 1-month locator check-in; (c) contacting participants by mobile phone within 24 h of missed visit; (d) attempting home visits if contact by mobile phone was not successful; and if the participant has changed address, the information will be solicited (while maintaining confidentiality) from locator contacts provided by the participant. A participant will be considered lost to follow-up if five phone contacts and home visits are unsuccessful.

## 3 Assessment of the preliminary effectiveness of Treat4All

### 3.1 Data analysis

We will first explore the distribution of continuous and categorical baseline variables using descriptive statistics and graphical representations, as appropriate (60). We will then conduct intent-to-treat comparisons between arms, using a log link and binomial error distribution to compare the proportion of heavy drinking days and engagement in HIV prevention or treatment (co-primary outcomes). For the proportion of heavy drinking days, we will estimate the self-reported percentage of heavy drinking days measured by the TLFB at 6 months. We will evaluate the extent to which mean values differ across groups using the deviance χ^2^ (61). As the effect of the intervention may differ by age and sex, effect modification by these factors will be examined. For successful engagement in HIV prevention or treatment, the design effect introduced by participant randomization will be addressed using a robust variance estimate. Probability ratios (PR), analogous to prevalence ratios at a specific time point, with 95% confidence intervals, will be used to estimate and compare the proportion of participants in each arm who achieved the outcome (successful engagement in HIV prevention or treatment) at 6 months. All analyses will also be sex-stratified to explore differences by sex to inform power calculations for a future implementation effectiveness trial. A significance level of 0.05 and two-sided 95% CIs will be used with no adjustment for multiple testing (62).

Additional analyses will assess self-reported ART adherence (among PWH), biologically confirmed HIV incidence (among HIV-negative participants), PrEP use (among HIV-negative participants), viral suppression (among PWH), and biologically confirmed STIs (among all participants). We will also assess the possibility of selective differential attrition across the arms at each post-baseline visit. We will estimate and compare an *a priori* set of baseline demographics by the study arm. If an imbalance is found, we will produce post-baseline regression-adjusted outcome estimates by recalibrating the baseline distribution of that group to the baseline distribution across all groups combined (63).

If >10% of observations are missing for a particular endpoint, we will quantify the sensitivity of our results to our assumption that all missing values have not achieved the outcome of interest (i.e., they did not decrease heavy drinking or did not achieve HIV outcome). We will assess predictors of loss to follow-up and apply inverse probability of observation weights (IPOW) to assume that data are missing at random (MAR). When disseminating the results, we will present weighted and unweighted results and a description of the accompanying assumptions.

### 3.2 Power considerations and sample size calculations

Our study will provide short-term efficacy data to inform a future, large-scale RCT. All tests will be based on a nominal 5% two-sided type I error, and confidence intervals will have a nominal 95% coverage. The main analysis on which our sample size calculation for the quantitative analysis was based is the short-term efficacy of the experimental intervention, Treat4All, vs. UC. Using two-tailed tests and *α* = 0.05, we will have >80% power to detect a difference as small as 16% points (e.g., 80% vs. 96%) with a sample of 144. To account for possible loss-to-follow-up/dropout of 10% based on our previous studies among patients receiving STI care in Malawi, we will inflate the sample size to 160 (*n* = 80 per arm [stratified 1:1 by HIV status]). While a sample size of 160 may limit generalizability, it is appropriate for this pilot study, which aims to assess feasibility and estimate effect sizes to inform a future, fully powered trial, in line with established guidance for early-phase research and prior pilot studies in similar contexts (64, 65). Sample size calculations were conducted using SAS 9.4, which will also be used for all statistical analyses.

## 4 Assessment of the implementation outcomes of Treat4All

Using mixed methods, we will assess the acceptability, feasibility, and fidelity of Treat4All to reduce heavy drinking and improve HIV prevention and treatment outcomes in patients receiving STI care who report heavy drinking in Malawi.

Acceptability will be measured at the participant level. Participant acceptability will be assessed with a 15-item pragmatic acceptability subscale developed by the Applied Mental Health Research [AMHR] Group at Johns Hopkins University and implemented with participants allocated to Treat4All (66). This instrument is a subscale of a validated measure developed to assess implementation domains for mental health or substance use interventions implemented in resource-limited settings. Participant acceptability will also be assessed via qualitative interviews to contextualize the acceptance of the intervention procedures. At the 3-month follow-up visit, we will purposively select a subsample of intervention participants (*n* = 10 who scored intervention acceptability high on the AMHR instrument; *n* = 10 who scored low intervention acceptability). The focus of patient interviews will be to understand the experience of alcohol reduction treatment to identify key attributes that facilitate or hinder acceptability and ways to improve implementation.

Feasibility will be assessed at the participant and interventionist level. The participant feasibility will be assessed using two quantitative measures. First, a 14-item pragmatic feasibility subscale of the AHMR measure will be implemented with participants allocated to Treat4All (66). Second, patient feasibility will be assessed by measuring the proportion of participants assigned to each intervention arm who will be enrolled and the proportion of enrolled participants attending all intervention sessions. Interventionist feasibility will be assessed by measuring intervention fidelity, the time and training required to achieve excellent fidelity, and the number of interventionists who leave the clinic or the study and need to be replaced.

Fidelity will be used to assess interventionists via independent raters and provider self-report. All intervention sessions will be audio recorded. A random sample (20%) of recordings will be assessed for fidelity. The independent rater will listen to these intervention sessions, translated into English, and complete the Yale Adherence and Competence Scale (YACS). At the end of each randomly selected session, the interventionists will complete an identical self-reported YACS. Fidelity assessment will assess adherence and competence.

### 4.1 Semi-structured interviews with interventionists and STI care providers

We will conduct semi-structured interviews with interventionists and STI care providers who have implemented Treat4All (*n* ~ 10). These interviews will occur 6 months after the intervention implementation has begun to allow for adequate experience in implementing the intervention. These interviews will address providers’ experiences with alcohol reduction interventions, attitudes toward substance use issues, willingness to incorporate alcohol reduction care into their practice, and perceptions about the acceptability, feasibility, sustainability, and effectiveness of the intervention. We will also conduct qualitative interviews with clinic administrators (*n* ≈ 2) focused on the feasibility and acceptability of incorporating alcohol reduction EBI into their clinics and the perceived importance of such interventions.

### 4.2 Quantitative data analysis

We will use descriptive analyses, such as measures of central tendency and dispersion (e.g., means and proportions), to summarize primary implementation outcomes: feasibility and acceptability. Feasibility will be measured as the proportion assigned to the intervention who agree to enroll and complete all sessions and the 14-item feasibility subscale. Acceptability will be measured using a 15-item acceptability subscale. We will also assess the extent to which participant-level factors are associated with acceptability and feasibility, including sociodemographic characteristics (age and sex), severity of baseline alcohol use, and baseline HIV status.

### 4.3 Qualitative data analysis

Qualitative interviews with study participants, providers, and administrators will be audiotaped, transcribed, translated, and coded for analysis using ATLAS.ti. The number of interviews will be determined by “theoretical saturation,” the point at which no new themes emerge within the interviews and similar information about key domains of interest is repeated by participants across interviews (67, 68). Transcript analysis will begin when data are collected to incorporate topics for further exploration into ongoing fieldwork. A research assistant and the principal investigators will independently complete line-by-line coding of the first five transcripts and meet to compare and contrast transcript coding, reach a consensus about codes and their definitions and applications, and build a preliminary codebook. We will meet weekly to discuss the coding process, resolve problems, ensure consistency, and decide whether the interview guide or sample should be adjusted in response to emerging findings. Qualitative data analysis consists of searching for patterns in the data and conceptualizing ideas that help explain the presence of those patterns (69). The analysis of semi-structured interview data will involve five steps: (1) reading for content, (2) deductive and inductive coding, (3) data display to identify emerging themes, (4) data reduction, and (5) interpretation. Memos will be written for each code. The codes will be refined during the analysis process. Responses will be compared within and across the participant groups.

### 4.4 Data triangulation

All data sources will be analyzed and triangulated to robustly understand the effectiveness and implementation of Treat4All. We will identify the contextually relevant approaches to successful implementation and practical difficulties in adoption and maintenance to inform future trials. Qualitative data enrich the quantitative findings and are optimal for gaining insights into the preliminary efficacy and implementation of Treat4All (70, 71). Qualitative data help us interpret quantitative findings and discover novel hypotheses to test for the future full-scale RCT testing the effectiveness of Treat4All to improve HIV prevention and treatment and alcohol outcomes.

## 5 Ethical considerations and trial management

A data monitoring committee was not established, as this trial involves training health providers on how to work in non-stigmatizing, person-centered ways with patients. Any serious adverse events will be reported to the ethics committee and study sponsor.

Participant information will be collected through quantitative surveys and laboratory data and stored in password-protected databases at UNC Project in Lilongwe. Data will be backed up daily, with secure on-site storage and restricted access. All files will be retained for five years and then destroyed. Confidentiality is ensured through staff training, private study procedures, and secure data handling. Data will be anonymized, encrypted, and accessible to authorized personnel only. Any serious adverse events will be reported to the ethics committee. Data sharing will occur routinely with the NIAAA-sponsored data repository, the NIAAA Data Archive (NIAAA_DA_), and more widely only after study completion, with strict quality control and confidentiality protocols in place throughout.

### 5.1 Role of trial sponsor and funder

This pilot implementation trial was funded by the National Institute on Alcohol Abuse and Alcoholism (NIAAA) grant (No. R34AA030939) awarded to Drs. Parcesepe and Lancaster. The funders play no role in the study design, data collection, management, analysis, and interpretation of data, writing of the report, and the decision to submit the report for publication.

## 6 Discussion

Our protocol outlines how our study will address critical priorities in the fields of alcohol use, HIV prevention and treatment, and implementation science. First, we will implement a systematically braided, culturally tailored alcohol reduction and prevention intervention using an HIV status-neutral approach. Project TrEAT is an effective, culturally appropriate, scalable EBI for alcohol reduction that has been primarily developed and implemented among PWH—but has not been adapted for HIV prevention or examined for HIV-status neutral outcomes (35–41). We will examine the preliminary evidence on the effectiveness of integrating TrEAT with HIV status-neutral counseling (Treat4All) to reduce heavy alcohol use and improve HIV outcomes among both PWH and those at risk for HIV. This approach moves beyond traditional HIV-focused interventions, offering a dual approach for both prevention and treatment.

Our study will also reach a broader patient population by targeting people who are not engaged in HIV care or prevention but are receiving treatment for STIs—a population often underserved yet at high risk because of heavy alcohol use. This is particularly important because those who consume alcohol heavily are more likely to acquire STIs, progress to alcohol dependence, and experience poorer engagement with HIV services. By focusing on those receiving STI care and who report heavy drinking, we will tap into a novel and crucially underserved population.

A key innovation of our study is the incorporation of alcohol biomarkers to complement self-reported alcohol use data. We will use PEth, a biomarker that objectively measures alcohol consumption over the past three weeks, to counteract the bias often associated with self-reported data. Few studies have explored how alcohol reduction interventions impact PEth levels (19, 49, 71), making this a unique aspect of our research that will provide more accurate insights into alcohol consumption behaviors. We will use PEth to objectively examine the differences in alcohol use between the two study arms.

To enhance the understanding of intervention acceptability, a purposive subsample of 20 participants—those with high and low acceptability scores—will be followed up qualitatively at three months to explore experiences that may not emerge through quantitative measures alone, though findings from this small, non-random sample may not be generalizable and should be interpreted with caution. All materials were carefully translated and back-translated into Chichewa, with bilingual experts resolving discrepancies and research staff conducting role-play exercises to ensure cultural and linguistic accuracy. Despite these efforts, subtle misinterpretations may persist, and participants’ responses may be influenced by these nuances.

Sustaining behavior changes is critical for long-term impacts. While this pilot trial focuses on short-term efficacy, the intervention was designed to be scalable and feasible for integration into routine care, thus supporting future implementation. To promote lasting engagement and adherence, future studies should incorporate booster sessions, mobile health support, or peer-based strategies (64, 72). These enhancements will help to assess the durability of the effects and guide long-term approaches to HIV prevention and alcohol reduction.

Once data collection and analysis are complete, we will publish the results and update the findings on ClinicalTrials.gov within one year of the study’s primary completion. We will also share the outcomes with relevant stakeholders, including the National AIDS Commission and the Ministry of Health, and disseminate the findings to clinical providers, patients, and staff through forums and conferences. The results will be presented at both local and international scientific conferences and submitted for publication in peer-reviewed journals. These findings will play a critical role in shaping future HIV prevention programs and combination trials in individuals with heavy alcohol use in Malawi.

Our study will adapt and pilot an alcohol reduction EBI for those receiving STI care who report heavy drinking to examine the short-term efficacy and implementation outcomes. Although we are unable to include a cost-effectiveness analysis in this phase due to resource constraints, we plan to incorporate this in a future RCT. While the current study will assess efficacy up to six months post-enrollment, a future trial will extend the analysis to 12 months. Additionally, while daily oral PrEP is already available in Malawi, we are well-positioned to integrate newer prevention methods, such as long-acting injectables and intravaginal rings, as they become available, ensuring that our study’s findings remain relevant for evolving HIV prevention strategies.

In summary, our study addresses the urgent need for effective, integrated interventions for PWH and individuals at risk of HIV who report heavy drinking. By leveraging our strong, robust infrastructure, we will produce a systematically braided, culturally tailored HIV status-neutral intervention for alcohol reduction and HIV prevention to evaluate in future effectiveness-implementation trials. Our study will directly contribute to expanding alcohol reduction and HIV programs across sub-Saharan Africa and other regions, where alcohol plays a key role in the HIV epidemic.

## Glossary

AIM: Assess, Identify, Make a Plan
AMHR: Applied Mental Health Research
ART: Antiretroviral Therapy
AUDIT: Alcohol Use Disorder Identification Test
DBS: Dried Blood Spots
EBI: Evidence-based intervention
HIV: Human Immunodeficiency Virus
NIAAA: National Institute on Alcohol Abuse and Alcoholism
PEth: Phosphatidylethanol
PrEP: Pre-exposure prophylaxis
PWH: People with HIV
RCT: Randomized controlled trial
sIMB: Situated Information, Motivation, and Behavior framework
SSA: Sub-Saharan Africa
STI: Sexually transmitted infections
TLFB: Timeline Followback
TrEAT: Trial for Early Alcohol Treatment
UNC: University of North Carolina at Chapel Hill
UC: Usual care
YACS: Yale Adherence and Competence Scale
